# Two-dimensional van der Waals heterostructures (vdWHs) with band alignment transformation in multi-functional devices

**DOI:** 10.1039/d2ra03439e

**Published:** 2022-11-02

**Authors:** Nasir Shehzad, Shahzad Saeed, Ismail Shahid, Imad Khan, Imran Saeed, Juan Antonio Zapien, Lixin Zhang

**Affiliations:** School of Physics, Nankai University Tianjin 300071 People's Republic of China; Department of Physics, Rawalpindi Women University Rawalpindi 43600 Pakistan shehzadsaeed2003@yahoo.com; Department of Materials Science and Engineering, City University of Hong Kong Hong Kong SAR PR China; School of Materials Science and Engineering, Computational Centre for Molecular Science, Institute of New Energy Material Chemistry, Nankai University Tianjin 300350 PR China; Department of Physics, University of Malakand Chakdara, Dir (Lower) 18800 KP Pakistan; Institute of Basic Sciences, Centre for Soft and Living Matter, Ulsan National Institute of Science and Technology (UNIST) Ulsan 44919 Republic of Korea

## Abstract

Two-dimensional van der Waals heterostructures (vdWHs) with tunable band alignment have the potential to be benignant in the development of minimal multi-functional and controllable electronics, but they have received little attention thus far. It is crucial to characterize and control the band alignment in semiconducting vdWHs, which determines the electronic and optoelectronic properties. The future success of optoelectronic devices will require improved electronic property control techniques, such as using an external electric field or strain engineering, to change the electronic structures directly. Herein, we review heterostructures fabricated as transition metal dichalcogenides (TMDCs) as one of their constituent monolayers with other notable 2D materials that can transfer from type-II to type-III (type-III > type-II) band alignment when a biaxial strain or electric field is applied.

## Abstract 2

Two-dimensional van der Waals heterostructures (vdWHs) with tunable band alignment have the potential to be benignant in the development of minimal multi-functional and controllable electronics, but they have received little attention thus far.

## Graphical abstract

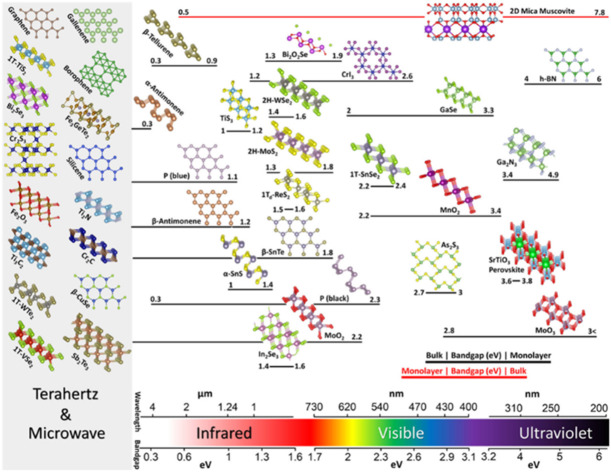

## 1. Introduction

van der Waals heterostructures (vdWHs) are atomically thin two-dimensional (2D) layered materials such as graphene or (TMDCs) that effectively enable the adjustable junction of different 2D materials at the atomic scale; producing a powerful and flexible tool for a broad range of electronics and optoelectronic devices.^1–14^ Physically layering 2D materials with incredible performance and reliability that use a weak vdW force (6.409 × 10^−21^ to 1.122 × 10^−20^ N m) has been coined initially as vdW heterostructures.^15^ Mixed-dimensional vdW heterostructures with both structural and material variations, such as 0D/2D (quantum dots),^16^ 1D/2D (nanowires),^17^ and 3D/2D (bulk),^18^ have paved the way for developing promising applications like light-emitting diodes (LEDs), transistors, oscillators, optical modulators, photodetectors, and other optoelectronics and passive components. One of the uttermost phenomenal characteristics of such heterostructures is band structure alignment, divided into three categories: straddling type I, staggered type II, and broken-gap type III.^19^ In addition, each type of band alignment is associated with a specific device application (discussed later). vdWHs have gotten much attention recently, however, the majority of vdWHs observed so far have band alignment of type-I or type-II, with just a rare vdWHs having a band alignment of type-III, significantly limiting its applications in tunnel field-effect transistors (TFET).^20^

2D semiconducting materials have the potential to be functional building blocks for vdWHs. Even though graphene (a single sheet of graphite) has been known for many years, it was considered that crystalline 2D materials, such as graphene, could not exist in nature due to thermodynamical instability.^21^ In 2004, researchers from Russia's Institute for Microelectronics Technology and the University of Manchester in the United Kingdom extracted a single layer from bulk graphite for the first time.^22^ Following the great success of graphene research, there has been a rush in research on various forms of two-dimensional (2D) atomic crystals.^22–24^ To circumvent the limitations posed by graphene's lack of a bandgap, a wide range of new 2D materials with notable physical and chemical features, such as TMDCs,^25–28^ phosphorene,^29–31^ hexagonal boron nitride (h-BN),^32,33^ and others, have been identified. The most striking examples of 2D materials are a family of layered transition metal dichalcogenides (TMDCs).^34^ TMDCs have a lengthy and successful track record. Linus Pauling, who discovered their structure in 1923, was the first to do so.^34^

By the late 1960s, about 60 TMDCs were known, with a minimum of 40 of them possessing a layered structure.^35^ In 1963, Robert Frindt published the first discoveries on using adhesive tapes to make ultrathin MoS_2_ films,^36^ and the first monolayer MoS_2_ suspensions were discovered in 1986.^37^ Semiconducting TMDCs have distinct properties that make them appealing as a conduit material in FETs, along with the absence of dangling bonds, structural stability, and mobility comparable to Si. The initial MoS_2_ research^25,38^ result proved that a viable FET could be made with a two-dimensional material, which motivated the community to seek more electrical devices. vdWHs have TMDCs monolayers as one of the constituents, with other notable 2D materials showing remarkable progress in FETS and optoelectronics fields.

Different band alignment heterostructures have unique properties and can be used in various applications. As a result, using an external electric field (*E*_field_) and biaxial strain can increase the application range of a single heterostructure.^39^ In two-dimensional materials, multiple-band alignment modulation is advantageous. The applied *E*_field_ and biaxial strain can effectively adjust the band alignment of the MoSe_2_/SnS_2_ heterostructures; a single material is used to modulate many alignments of bands.^39^ In 2D materials, one of the utmost vital means of tuning bandgaps stems from the fact that these materials can be stacked in any order, essentially free of the lattice-matching constraints that exist in conventional quantum well heterostructures.

Most importantly, due to their enormous surface-to-volume ratios, the band structure of 2D layers is hugely responsive to external interactions, meaning that external disturbances can significantly alter their bandgaps and electrical structure. Meanwhile, the quality of vdWHs is critical for producing a high-performance TFET, with sharp band edges and low defect density. TMDCs are promising materials in this context because of their sharp band edges and atomically uniform thickness.^40^ We review the impact of electric field and biaxial strain on the band alignment of heterostructures and discuss the physics of bandgap transformation [type II (III) > type III (II)] in vdW heterostructures constructed by TMDCs with TMDCs or other 2D materials for achieving multi-functional vdWHs. As previously mentioned, the band alignment of the heterojunction is an important parameter for archiving high-performance TFET and optoelectronic devices.

This review is organized as follows: initially, we present an overview of TMDCs and some other notable 2D materials structures with their bandgaps and vdW heterostructure fabrication. Reviewing type II and type III band alignments in various 2D vdW heterostructures materials and briefly explaining their most intriguing properties. The effect of strain engineering and the external electric field on the bandgap in van der Waals heterostructures are then discussed. Following that, we look at the most recent band alignment alteration in vdW heterostructures built with TMDCs monolayer. Finally, we will suggest future study targets for researchers based on previous literature.

## 2. Structure and bandgap of TMDCs and notable 2D family

**Fig. 1:**
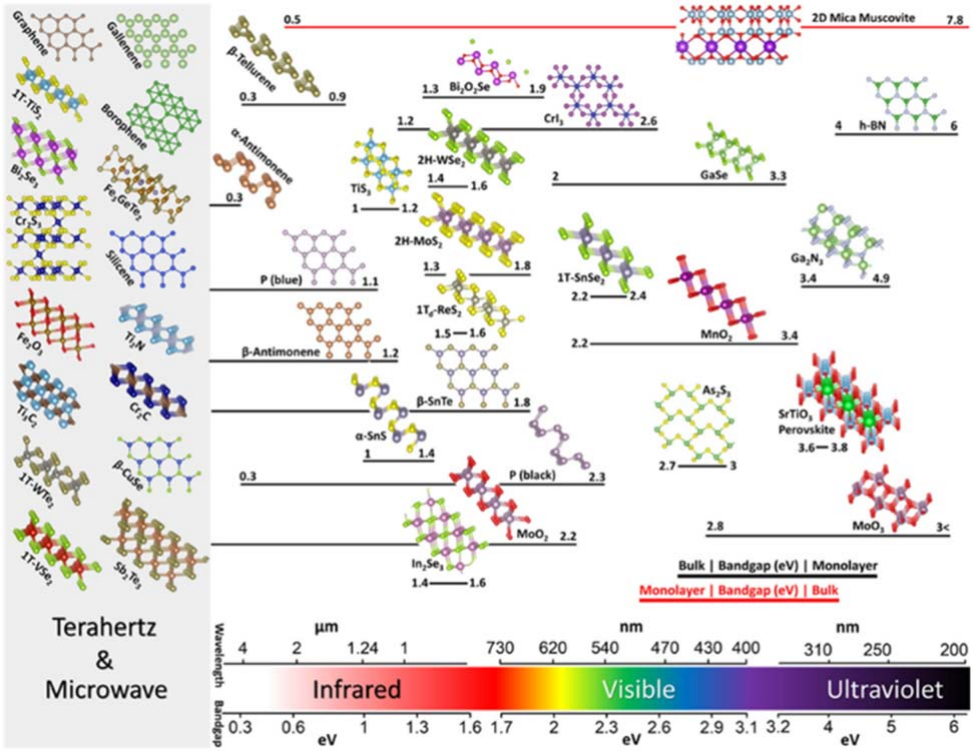
Shows the bandgaps of 2D materials for a selected family. The perspective view of the 2D materials with the crystal structures is preferred due to their experimental demonstration and significance. The bandgap/wavelength scale at the bottom guides the arrangement of the structures, while the bandgap range for each structure starting from bulk to monolayer is shown by the bar beneath. The bandgap for bulk is usually lesser than the bandgap for monolayer (black bars), but there are exceptions (red bars). The bandgap of zero or near-zero of metallic, or semi-metallic 2D materials is shown on the far left, denoted by a grey box. Reprinted from ref. 45 with permission from Nature, copyright 2020.

The crystal structures and bandgaps of 2D materials are presented in Fig. 1. Beyond graphene, research on 2D materials is already quite broad, generating enthusiasm for various uses.^41^ Following graphene,^24,42–44^ the monolayers of TMDCs were separated using comparable mechanical exfoliation techniques. MX_2_ (M = transition metals, X = S, Se, and Te) is the chemical composition of the TMDCs and exists in morphological phases such as 2H, 1T, 1T′, and 1Td. Semiconducting 2H TMDCs (*e.g.*, MoS_2_), in particular, has drawn a lot of consideration for their exciton physics and unique circular valley dichroism, with a bandgap range in the 1–2 eV.^46–48^ The TMDCs of T phases (for example, WTe_2_), on the other hand, are usually metallic to semi-metallic^49^ and are likewise intriguing for their topological characteristics^50^

These structural phases can also be viewed in terms of different stacking orders of the three atomic planes (chalcogen–metal–chalcogen) forming the individual layers of these materials. The 2H phases correspond to an ABA stacking in which chalcogen atoms in different atomic planes occupy the same position A, and are located on top of each other in a direction perpendicular to the layer. By contrast, the 1T phases are characterized by an ABC stacking order. Depending on the particular combination of transition metal (group IV, V, VI, VII, IX or X) and chalcogen (S, Se or Te) elements, the thermodynamically stable phase is either the 2H or 1T phase.

However, the other can often be obtained as a metastable phase. For example, for five of six possible chemically distinct bulk TMDCs formed by group VI transition metals (metal = Mo or W; chalcogen = S, Se or Te), the 2H phase is thermodynamically stable and the 1T phase can be obtained as a metastable phase. An exception is represented by WTe_2_, for which the stable bulk phase at room temperature is the orthorhombic 1Td phase. The structure of TMDCs is further defined by the stacking configuration of the individual layers in the case of multilayer and bulk samples and by possible distortions that lower the periodicity.

These distortions, if pronounced, can result in the formation of metal–metal bonds—as it happens, for example, in the dimerization of the 1T phase of group VI TMDCs, which results in the 1T′ phase, or the tetramerization of rhenium dichalcogenides such as 1T-ReS_2_.^5^ The 1T phase stands for a metallic phase, while the 2H and other phases are semiconducting. In particular, semiconducting 2H TMDCs (*e.g.*, MoS_2_) have semiconducting transition-metal trichalcogenide such as TiS_3_ with a crystal structure consisting of quasi-one-dimensional atomic chains of the stacked triangular prism,^51^ which is another chalcogenide-based 2D material.

Also 2D chalcogenides, such as GaSe containing group III elements,^52^ occur in various polytypes depending on their layer-stacking arrangement. Monochalcogenides that have puckered and buckled symmetries, such as SnS and GeSe,^53^ make up the group-IV elements' 2D chalcogenides, whereas their 1T dichalcogenides such as SnS_2_,^54^ are usually semiconducting. MXenes^55^ are a group of two-dimensional transition metal carbides, nitrides, and carbonitrides that have ceramic-like features such as metal-like electrical and thermal conductivity and structural stiffness.^56–59^ M_2_X, M_3_X_2_, or M_4_X_3_ are chemical formulae for them, within which M shows early-transition metal and X represents carbon or nitrogen, for example, Ti_3_C_2_. Ga_2_N_3_ and other III–V 2D semiconductors with large bandgaps have recently been created.^60^ Materials such as hexagonal boron nitride (h-BN), whose bandgap is large,^61,62^ are essential in 2D materials because of their ultra-flat and inert nature that enables them to be utilized in the form of substrate for high mobility 2D electronics.

Perovskites (for example, SrTiO_3_) have been investigated extensively as a material for solar cells^63–66^ and topological insulators (for example, Bi_2_Se_3_ and Sb_2_Se_3_) that are recognized for their spin-momentum-locked and topologically protected electrical transport,^67^ are two other examples of layered materials. Other famous examples of constituent 2D materials, aside from graphene, and can range from metallic to semiconductor materials includes phosphorus (for example blue and black phosphorus),^30^ silicene,^68^ germanene,^69^ tellurene,^70^ gallenene,^71^ antimonene,^72^ and borophene.^73^ The range of bandgap for the aforementioned 2D layered materials, starting from monolayer to bulk, cover the broad electromagnetic spectrum, including from the terahertz to ultraviolet, and are depicted in Fig. 1.

## 3. Fabrication of 2D heterostructures

Vertical or lateral heterostructures might be formed in 2D materials due to their layered structure. Different fabrication techniques can be used for heterostructures, depending on their topology. The fabrication procedures are divided into two groups here: mechanical stacking and direct synthesis.

### 3.1 Heterostructures by manual stacking

**Fig. 2:**
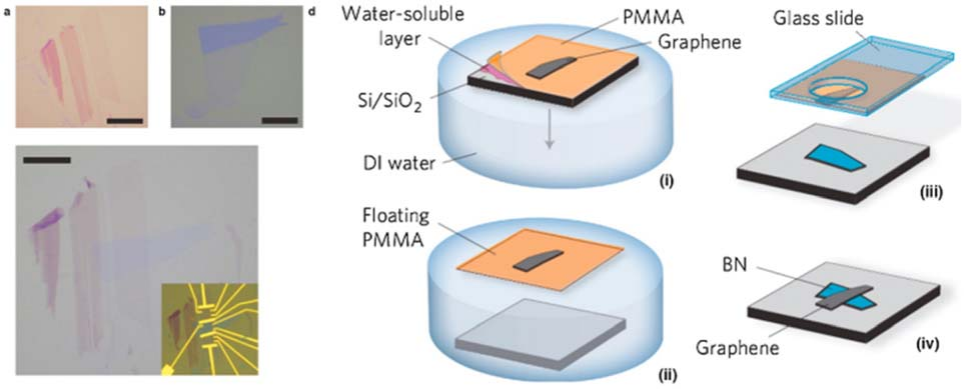
Shows the mechanical stacking: a schematic depiction of the optical view, the transfer and stacking process of the graphene/h-BN devices (scale bars, 10 μm).^61^ Reprinted from ref. 61 by permission from Nature, copyright, 2010.

2D flakes that are exfoliated chemically or mechanically can be deliberately layered to produce 2D heterostructures, with vdW force between the interlayer effectively holding the heterostructure, thanks to the introduction of exfoliation^22^ and transfer^61,74^ procedures for layered materials. Mechanical/chemical exfoliation methods can be used to generate 2D flakes from the bulk materials or they can be extracted directly on substrates from synthetic 2D layers. For the electrical and optical properties, the sequence of stacking and the interface of the heterostructures are critical. Due to the flexibility to generate different stacking lattice orientations, the interface of heterostructures has configurable physical features based on the two stacked materials interaction strength. Fig. 2 shows a graphene device on h-BN interface layers prepared *via* the mechanical stacking method by Dean *et al.*^60^

The atomic flat and nearly free charge trapping of h-BN layers serve as an excellent substrate for graphene. Graphene devices made on h-BN substrates have nearly order-of-magnitude greater carrier mobility than those made on amorphous SiO_2_ substrates. The reduced roughness, decreased doping, and better chemical stability of the graphene layers on h-BN demonstrate the crucial function of the interfaces. Other examples of heterostructures by manual stacking are graphene/MoS_2_ and MoS_2_/WSe_2_.^75,76^

Another method to fabricate vdWHs by mechanical exfoliation is the PDMS technique.

#### 3.1.1 PDMS deterministic transfer method

A viscoelastic stamp-based all-dry transfer method was developed by Castellanos-Gomez *et al.* Since no wet chemical steps involved in this technique, it is particularly favorable for fabricating devices with freely suspended 2D materials and for enhancing transfer speed.^77^

**Fig. 3:**
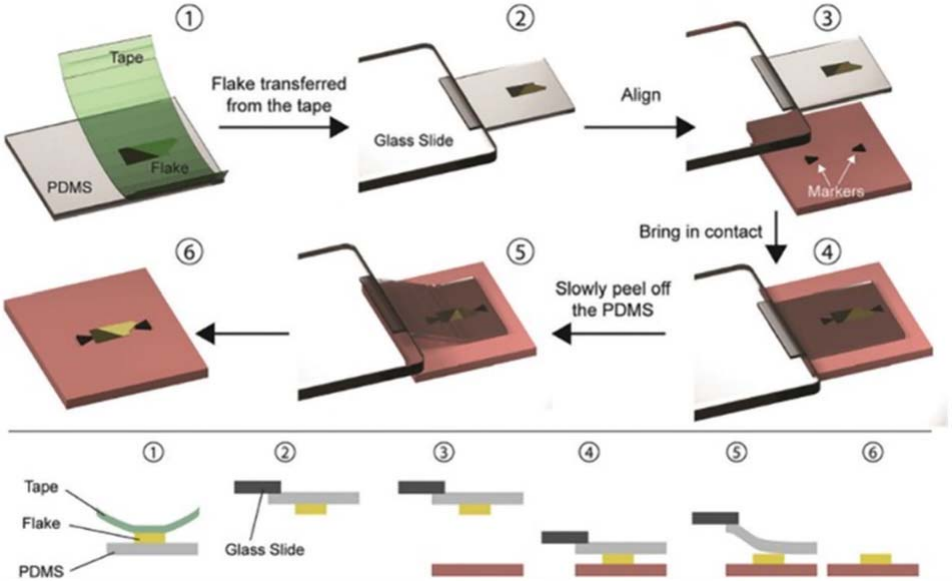
The PDMS dry transfer method. The flake to be transferred is exfoliated onto a PDMS stamp (1) and the stamp is then attached to a glass slide connected to a micromanipulator (2). Using a microscope, the flake can be aligned with the final substrate (3) and brought into contact (4). By slowly peeling the PDMS stamp (5) the flake can be deposited on the substrate (6). Reproduced from ref. 81 with permission from Royal Society of Chemistry (RSC), copyright 2020.

This method is illustrated in Fig. 3 and is based on the viscoelastic characteristics of PDMS (polydimethylsiloxane). Initially, a flake is mechanically exfoliated with Nitto tape onto a viscoelastic PDMS stamp that is readily available (Gelfilm by Gelpaks) (1). After that, the PDMS stamp is cantilevered and affixed to a glass slide that is coupled to a micromanipulator (2). One can perfectly align the flake on the PDMS with the target substrate (3) while the procedure is being observed under an optical microscope, and then bring the two into contact (4). The glass slide is carefully lifted so the PDMS can gradually detach from the substrate,^78^ releasing the flake from the PDMS stamp and leaving it in the desired location on the substrate (6).

The PDMS-based deterministic transfer approach is described here using ref. 77, but there are differences^79,80^ from the transfer method created by Castellanos Gomez and colleagues originally.^81^

### 3.2 Direct synthesis of 2D heterostructures

Although the exfoliation process can synthesize high-quality 2D crystals for fundamental research, however, controlling the layer number, location, and interface of the resulting heterostructures is still tricky, making practical production difficult. For TMDCs heterostructures, chemical vapor deposition (CVD) has recently been demonstrated to be a potential method for fabricating large domain 2D building blocks. It is also feasible to directly synthesize various 2D heterostructures with laterally stitched or vertically stacked interfaces. The lateral and vertical heterostructures produced by the CVD process are discussed below here separately.

#### 3.2.1 Vertically stacked 2D heterostructures

**Fig. 4:**
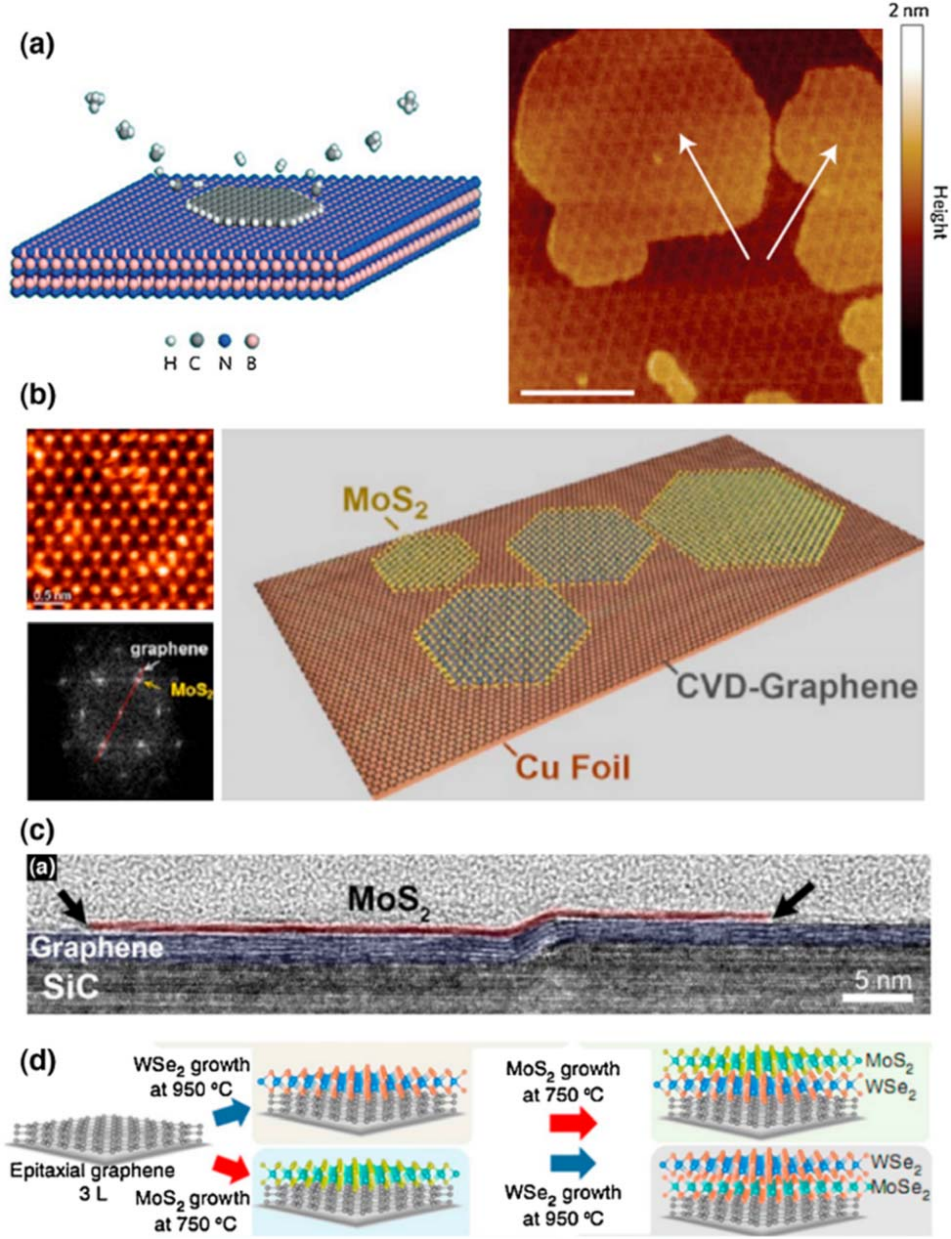
Shows the growth of 2D vertical heterostructures. (a) Illustration of the Morie pattern of the heterostructure and the plasma-assisted deposition process for graphene produced on h-BN. The graphene layer is indicated by white arrows (scale bars 100 nm).^82^ Reprinted from ref. 82 with permission from Nature, copyright 2013. (b) A TEM image of MoS_2_ grown over graphene, as well as a schematic depiction.^83^ Reprinted from ref. 82 with permission from American Chemical Society (ACS), copyright 2012. (c) HRTEM cross-section of a layered heterojunction of MoS_2_ and graphene on a SiC substrate step edge.^84^ Reprinted from ref. 84 with permission from American Chemical Society (ACS), copyright 2014. (d) MoS_2_/WSe_2_/graphene and WSe_2_/MoS_2_/graphene vertical heterostructures produced by oxide powder vaporization and MOCVD.^85^ Reprinted from ref. 85 with permission from Nature, copyright 2015.

CVD techniques can develop one 2D material on top of another, resulting in vertically stacked heterostructures. Because 2D layers have only vdW contact between them, therefore the mechanism is most likely vdW epitaxy, with the lattice mismatch being the most difficult hurdle to surmount. Early experiments begin with h-BN and graphene, two materials with identical lattice constants. On an h-BN substrate, Yang and colleagues^82^ demonstrated a plasma-assisted deposition technique for producing single-domain graphene (Fig. 4a). Graphene has a favored orientation on the h-BN lattice, the size of which is restricted only by the underlying h-BN area.

Shi and colleagues^83^ used the thermal breakdown of ammonium thiomolybdate precursors on graphene surfaces to build a vertically stacked MoS_2_/graphene structure (Fig. 4b). Even though MoS_2_ has a greater lattice constant than graphene, graphene is still a feasible growth substrate for MoS_2_. To accommodate the lattice mismatch, the strain may be involved in the formation of MoS_2_ on graphene. Lin and coworkers used CVD techniques to demonstrate the direct development of MoS_2_, WSe_2_, and h-BN on graphene (Fig. 4c),^84^ where the underlying graphene was grown epitaxially on SiC. Lin and coworkers, on the other hand, show that by employing oxide powder vaporization and metal–organic chemical vapor deposition (MOCVD) methods, they can directly synthesize MoS_2_/WSe_2_/graphene and WSe_2_/MoS_2_/graphene heterostructures *in situ* (Fig. 4d).^85^

At room temperature, the electrical transport of the heterostructure exhibits a sharp negative differential resistance (NDR) due to the unusual resonant tunneling of charge carriers at the interface. In mechanically stacked junctions, this observation has not been made. Although NDR can also be observed in mechanically exfoliated samples, however a low temperature down to ∼77 K is necessary for these studies. The tunneling is very sensitive to the interface charge perturbations, therefore, charge impurities and defects introduced during the transfer process may kill the NDR. The major difference here is for the directly synthesized samples; the interface is clean enough to allow the observation of resonant tunneling at room temperature. These direct syntheses can theoretically be applied to other materials, opening up new possibilities for heterostructures manufacturing. On the other hand, the thermodynamically dominated process may limit controllability in terms of stacking sequence, twisting angle, or domain size.

#### 3.2.2 Laterally stitched 2D heterostructures

**Fig. 5:**
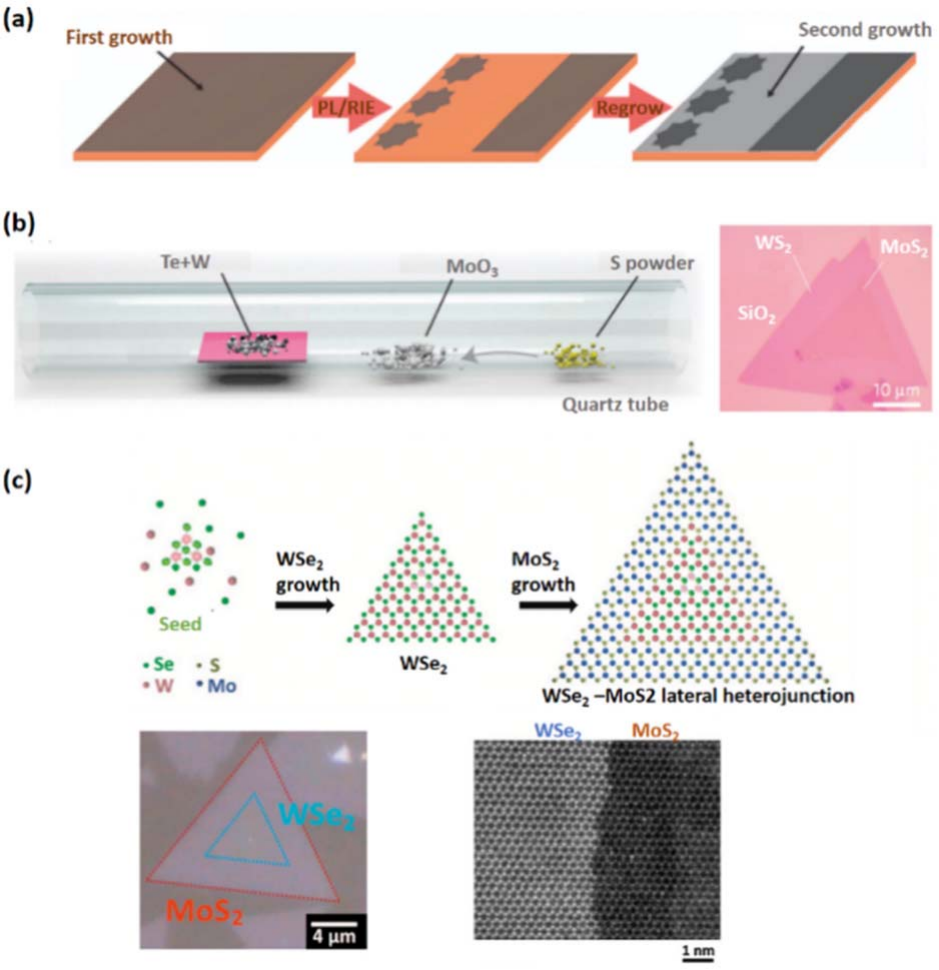
Shows the 2D lateral heterostructures grow in-plane epitaxially. (a) Schematic depiction of the CVD development process for graphene/h-BN lateral heterojunctions.^86^ Reprinted from ref. 86 with permission from Nature, copyright 2012. (b) Optical image of the WS_2_/MoS_2_ lateral heterostructures and schematic representation of the one-pot synthesis setup.^89^ Reprinted from ref. 89 with permission from Nature, copyright 2014. (c) The optical picture of the monolayer WSe_2_/MoS_2_ lateral heterojunction, as well as its two-step production. The comparable high-resolution STEM pictures taken at the interface are shown on the right.^90^ Reprinted from ref. 90 with permission from The American Association for the Advancement of Science (AAAS), copyright 2015.

Because covalent bonds exist in 2D materials, therefore only direct growth can produce lateral heterostructures. The graphene/h-BN lateral stitched heterostructures were constructed by CVD-growing h-BN over patterned graphene, which results in a continuous 2D heterostructures sheet, on the report of Levendorf and colleagues^86^ (Fig. 5a). The general challenge with lateral heterostructures is strain release between two materials. For the first time, one-pot synthesis was employed to exhibit lateral TMD heterostructures. For example, the lateral heterostructures WS_2_/WSe_2_ and MoS_2_/MoSe_2_ can be grown using a physical vapor transport approach with sequential source switching,^87^ a physical vapor transport method with mixed sources,^88^ and a one-spot CVD growth with multi-sources (Fig. 5b).^89^

Li and colleagues recently established a two-step CVD technique for epitaxial development of the WSe_2_/MoS_2_ lateral junction,^90^ in which WSe_2_ is first synthesized on the substrate through van der Waals epitaxy, and then MoS_2_ is edge epitaxial along the W growth front. The reaction status must be revealed during the second stage of the two-stage CVD procedure to avoid alloy formation (Fig. 5c).

## 4. Electronic properties of type II and type III band alignment and their properties

**Fig. 6:**
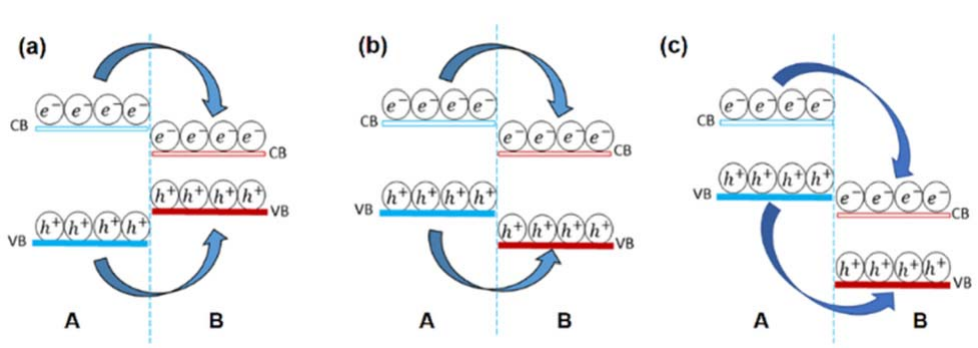
Diagram of band structure (a) type-I straddling band structure (b) type II staggered band structure (c) type III broken band structure.

Band alignment is the fundamental property of 2D heterostructures. The band structures, particularly the band alignment, are responsible for the majority of charge transport behavior and illuminance features. The band energy can be computed using the first principle theory and tested using μ-XPS. When two different materials with appropriate band gaps are combined to form heterostructures, three sorts of band alignments emerge type I, type II, and type III (shown in Fig. 6). In a type-I heterostructure (straddle gap), the conduction band minimum (CBM) and valence band maximum (VBM) are both present in the same material, as shown in Fig. 6a. Due to the charge carriers' well-spatial confinement and efficient reduction of undesirable excitons dissociation; photo-excited electrons and holes in the component with a wider bandgap tend to move to the component with a narrow bandgap under light illumination, significantly improving illumination intensity and efficiency.^91^ The type-I structure has been used to create a variety of unique optoelectronic devices.

The CBM and VBM in a type-II heterostructures are positioned in unlike components, resulting in a staggered gap, as shown in Fig. 6b. Spatial separation of electron–hole couples is produced by transporting the electrons and holes that have been stimulated by light in the opposite direction to CBM and VBM at discrete layers. Chen *et al.*^92^ used energy-state resolved ultrafast visible/IR micro-spectroscopy to study the charge transfer process in a MoS_2_–WS_2_ type-II heterostructure. The holes (basically electrons) migrate fast to WS_2_ (MoS_2_) after photo-excitation, forming interlayer hot excitons, which are high-energy intermediate states of electron–hole pairs. Because of the enhanced energy, the separation of the interfacial hot excitons occurs easily by the internal/external potentials and contributes to the photocurrent, resulting in a wide electron–hole distance and low binding energy. The creation of intralayer hot excitons (600 fs to 2.0 ps) is significantly faster than the formation of interlayer hot excitons (greater than 50 fs), which is quite interesting.^92^ The usage of type II heterostructures in photoelectric conversions, such as photovoltaics and photodetectors, is theoretically advised by such unequivocal experimental findings.

When the CBM is much lower compared to VBM, the band alignment is characterized as type-III (broken gap, Fig. 6c) premised on type-II. In broken gap we can see the CB of material “B” is lower than the VB of material “A” which may be because of the difference between electron affinities of contacting materials. Because of their wider band offset, type-III heterostructures make interlayer charge transfer easier, as evidenced by the strong PL quenching events. These transported holes and electrons aggregate in multiple layers on each side of the heterojunction interface to generate highly doped states, allowing electrons to tunnel directly between two materials *via* interlayer recombination when pushed by an external electrical field. This method resulted in the creation of vdW Esaki tunnel diodes with strong negative differential resistance (NDR) phenomena, which might be used in multi-valued logic applications and radio frequency oscillators.^93–97^ Furthermore, this kind of band-to-band tunneling (BTBT) is predicated on a cold charge injection technique that can be used to make tunnel field-effect transistors (TFETs) having lower power consumption and lower subthreshold swing values (60 mV dec^−1^).^98–100^

## 5. Strain and electric field effect

In practice, manipulation of the electronic characteristics of nanomaterials and nanodevices is accomplished *via* the electronic field.^101^ External factors such as in/out-plane strain and external electric fields can also influence the ideal electrical properties of the vdWHs.^102^ From a macro perspective, the procedure of infusing mechanical energy into a system is the procedure of introducing strain to a 2D material. This energy is trapped inside the material within the elastic deformation limit, resulting in a non-equilibrium condition that leads to a series of fundamental property changes. The energy must be released through atom reconstruction, phase transition, or fracture if the elastic limit is surpassed. From an atomic perspective, strain alters the original state of atomic bonding by elongating or shortening chemical bond lengths and affecting lattice symmetry. As a result, the material's electrical structure changes, as do several physical properties. Because the strain engineering effect is provided directly on the lattice, it's easy to see why it is such a simple and ubiquitous approach.^103^ The strain is computed using the formula (*a* − *a*_0_)/*a*, where *a*_0_ and *a* are the unstrained and strained heterojunction lattice properties, respectively. Strain changes the crystal lattice which influencing the band structure. The volume change shifts the positions of energy bands and the lowering of the crystal-symmetry splits degeneracies in bands.

**Fig. 7:**
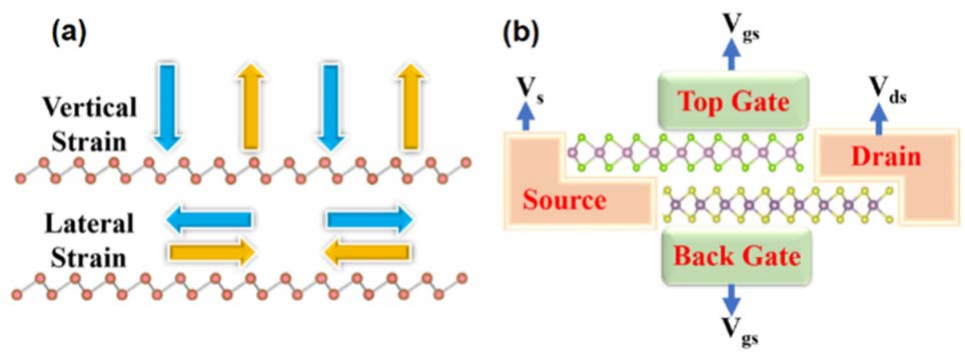
Schematic figure of (a) showing in/out of plan strain on heterostructures (b) a prototype of a multi-purpose device based on heterostructures.

More than 5% tensile strain in MoSe_2_/SnS_2_ heterostructures can cause the band alignment to shift from type I to type III.^104^ In/out of plan strained heterostructures are seen schematically in Fig. 7a. Practically, adding an external electric field has the benefit of being simple to achieve and manage, as well as being environmentally benign. The impact of electric fields on the electrical characteristics of heterostructures has piqued researchers' attention in both basic and applied research. When an external electric field pushes electrons and holes in opposite directions, the energy band boundaries in a semiconductor are deformed in real space. In 2D semiconductors, the usage of the electric field can control the locations of VBM and CBM. The re-distribution of charge in heterostructures is proposed to explain the potential mechanism of band gap modulation under electric fled. The charge density difference is usually to elucidate charge transition information, bonding structure, and bonding strength during the bonding process of atoms in layers. Because of the quantum-confined Stark effect,^105–107^ the electrons would transfer from one side to the other side of atomic layers against the direction of the electric field, leading to an overall net shifting in energy. Due to the asymmetry of bonding between atoms, spontaneous electric polarization exists along the *c*-axis. Thus, the equivalent field is enhanced or weakened, as the electric field is along or against the *c*-axis direction, which results in to modulate of the electronic band structure and increases or decreases the band gap increases due to the Stark effect.^105^

For example, when a perpendicular external electric field is applied on the surface of a monolayer GaGeTe, the CBM shifts to a low energy state.^108^ In 2D heterostructures, the electric field can also modify the band alignment.^109,110^ When the electric field is stronger than V Å^−1^, a type II band alignment in MoTe_2_/WSe_2_ heterostructures converts to a type III band alignment.^111^ We presented a vdWH-based multi-purpose device prototype, which is depicted in Fig. 7b. The device consists of a source, a drain, a channel, and two gates for injecting the external electric field. When the gate voltage is negative, this device with type-III band alignment can be utilized for tunnel field-effect transistors, and when the gate voltage is positive, this device with type-II band alignment can be used for electrical applications. The versatility of such a device requires more experimentation.

## 6. The band alignment transformation in TMDCs heterostructures

**Fig. 8:**
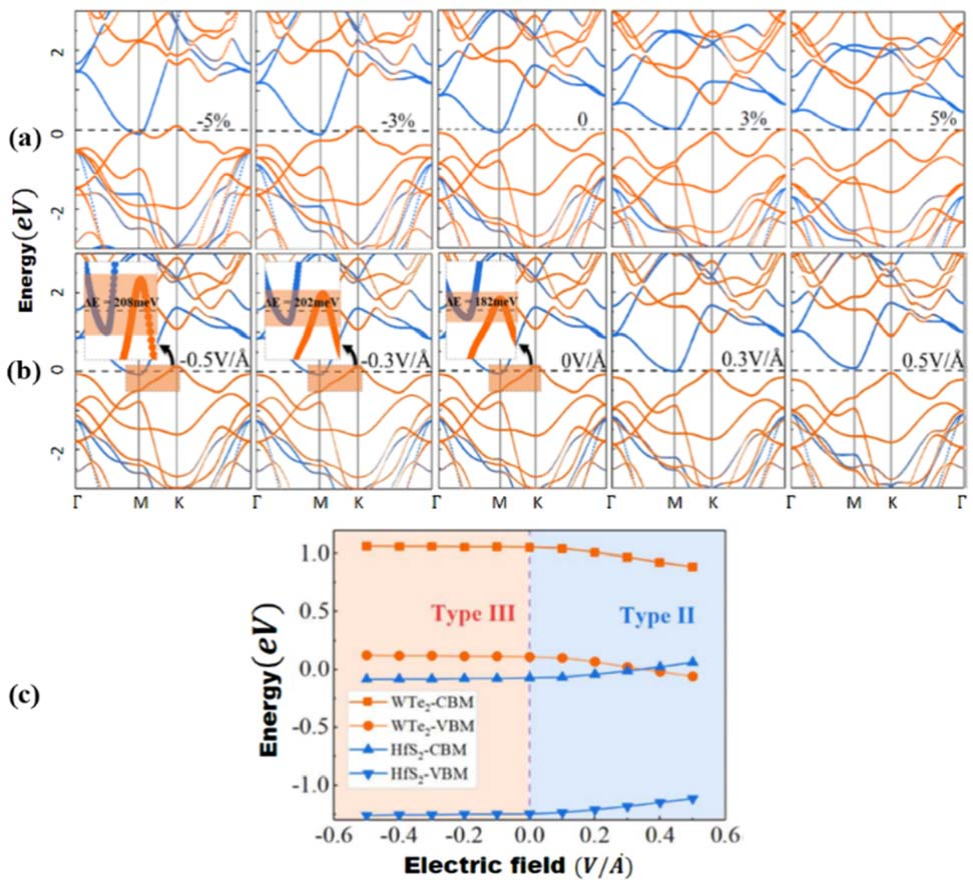
(a) WTe_2_/HfS_2_ vdWH band topologies under various biaxial strains, with orange and blue lines indicating contributions from the WTe_2_ and HfS_2_ layers, respectively. 0 eV is selected as the Fermi level. (b) Under electric fields, band structures of WTe_2_/HfS_2_ vdWH, with the orange and blue lines indicating contributions from the WTe_2_ and HfS_2_ layers, respectively. (c) WTe_2_ and HfS_2_ layer band edge positions in WTe_2_/HfS_2_ vdWHs under varied electric fields. Reprinted from ref. 20 with permission from American Chemical Society (ACS), copyright 2019.

**Fig. 9:**
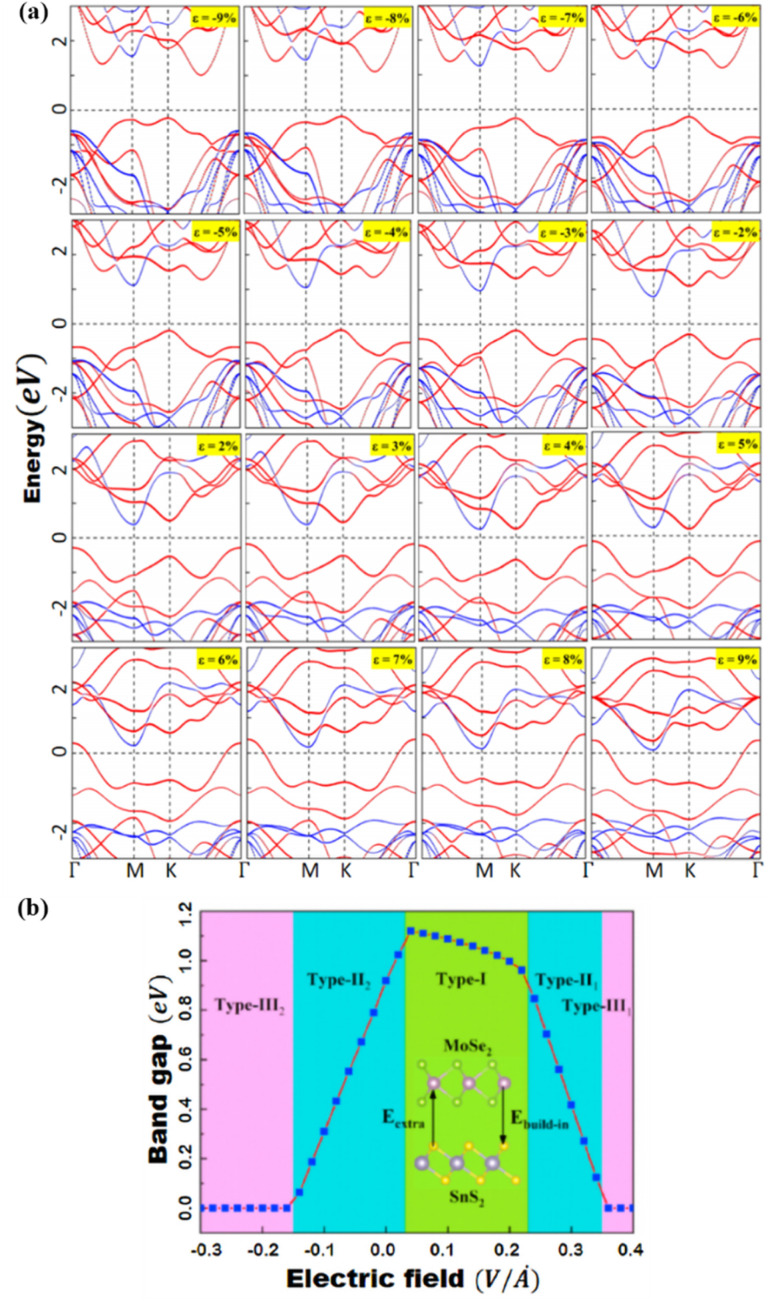
(a) Under pressure, the band structures of MoSe_2_/SnS_2_ heterojunctions. MoSe_2_ and SnS_2_ are represented by the red and blue lines, respectively. (b) The bandgap of MoSe_2_/SnS_2_ heterojunctions varies as the external E-field changes. Reprinted from ref. 20 with permission from Elsevier, copyright 2021.

Peripheral perturbations through an external electric field and bi-axial strain can be used to transform band alignment from type II to type III or from type III to type II in 2D heterostructures formed by TMDCs with other 2D materials, allowing these vdWHs to be used as multifunctional materials. In this part, we'll explore several recent 2D heterostructures with external electric fields or strain-influencing band alignment. We will look at how the changes transpire and utilize that data to generate future predictions. Because of the presence of band-to-band tunneling, WTe_2_/HFS_2_ heterostructures have broken band alignment (type III) and may display a noticeable negative differential resistance phenomenon (BTBT).^104^ The band topologies of WTe_2_/HfS_2_ vdWHs are illustrated in Fig. 8a under various biaxial strains. The WTe_2_/HfS_2_ vdWHs band structure shifts from band alignment of type-III to type-II under tensile biaxial strain. It can be seen that the CBM of HfS_2_ rests comparatively below, the Fermi level under >3% strain. WTe_2_/HfS_2_ vdWHs with the band alignment of type II can be used in solar energy conversion systems and a variety of optoelectronics applications. The electric field can also affect band alignment, as seen in Fig. 8b and c, which shows the band alignment transition in WTe_2_/HFS_2_ heterostructures. When provided a positive electric field, it dragged down the band edges of the WTe_2_ layer while in the case of the HfS_2_ layer are dragged up, resulting in the fascinating transition from type-III to type-II band alignment. The built-in electric field is weakened when a positive electric field is applied to WTe_2_/HfS_2_ vdWH an observable transition between the band alignments of type-III and type-II can be seen.^20^ Under varied electric fields and in-plain biaxial strain, type-II and type-III band alignment may be accomplished, extending the MoSe_2_/SnS_2_ heterostructures application range. As shown in Fig. 9b, the electric field can achieve all three types of band alignment in MoSe_2_/SnS_2_ heterostructures: type-I (0.03 < *E* < 0.23 V Å^−1^), type-II 1 (0.23 < *E* < 0.35 V Å^−1^), type-II 2 (−0.15 < *E*) and type-III 2 (*E* < −0.15 V Å^−1^). When a tensile strain of more than 5% is applied to MoSe_2_/SnS_2_ heterostructures, the band alignment transforms from type-I to type-III (Fig. 9a).^39^

Honglin *et al.*^112^ showed that increasing strain in the BP–MoTe_2_ heterostructures from 3.1% to 4.8% causes the CBM and VBM of the BP sublayer to fall below those of the MoTe_2_ sublayer, resulting in type-III from type-II band alignment. When the strain in the BP–WTe_2_ heterostructures is increased from 4.8 percent to 8.8 percent, the type-III band alignment is created for the lower CBM and VBM of the BP sublayer compared to WTe_2_. Furthermore, Yuxiao *et al.*^113^ reported that band alignment shifts from type-II to type-III for an electric field higher than 0.6 V Å^−1^ is given to the MoTe_2_/WSe_2_ heterostructures. In MoS_2_/WSe_2_ heterostructures, a greater electric field is required to convert the type-II band alignment to type-III, whereas in GeC/WS_2_ heterostructures, a large biaxial strain of 8% is required to adjust the type II to type III transition, as reported in ref. 110 and 114. Furthermore, a positive electric field can transform the band alignment from type-II to type-III in the heterostructures composed of main-group metal chalcogenides such as in Ges/SnS_2_.^101^

To demonstrate how a multifunctional device may be constructed employing an external electric field and strain, we studied and examined various examples from the ocean of TMDCs-based heterostructures constructed with another TMDCs monolayer or other 2D materials. We have included a few heterostructures that exhibit multifunctional device behavior. The 2D(TMDCs)/TMDCs(2D) heterostructures are predicted to expand the building blocks for multifunctional applications by providing unrivaled characteristics and performance. As a result, it's thought that putting more effort into this intriguing subject might pave the way for the next generation of contemporary electronics and optoelectronics to be realized at the nanoscale.

Note: several factors can tune the properties of 2D heterostructures such as interlayer distance, doping, defects, *etc.*, which we will discuss in detail in our forthcoming review paper. Herein we discussed the two simples to achieve and understand external perturbations *i.e.*: strain and electric field.

## 7. Conclusion and perspective

The burgeoning field of 2D material research has sparked a lot of interest in exploring vdW heterostructures made of various materials. Integration of ultrathin 2D layers with TMDCs functional materials is one of them, and it offers new attractive candidates for electrical and optoelectronic applications. The methods of synthesis and proof-of-concept gadgets of 2D(TMDCs)/TMDCs(2D) heterostructures are methodically reviewed here. For constructing the heterostructures, various mechanical transfers and direct synthesis by bottom-up procedures have been reviewed. In the meanwhile, the band structure alignment of a standard 2D(TMDCs)/TMDCs(2D) heterojunctions is examined. 2D(TMDCs)/TMDCs(2D) heterostructures applications in FETs, photodetectors, photocatalysts, and gas sensors are also discussed.

In this study, several hybrid heterostructures have exhibited widespread use of 2D(TMDCs)/TMDCs(2D) combinations, which are desired for producing high-performance practical multi-functional devices. 2D heterostructure semiconductors provide a novel platform for studying band structure engineering effects. The state-of-the-art bandgap engineering techniques were discussed here, although their applications in materials science and multi-functional devices are still in the early phases. For both basic research and real-world applications of 2D(TMDCs)/TMDCs(2D) heterostructures, there are several problems and possibilities, which are succinctly outlined here.

(i) When the biaxial strain and electric field are applied to the band structure generated by heterostructures comprising one or both monolayers as TMDCs, an intriguing transition of band type occurs. The change of a heterostructure's band structure from type II (III) to type III (II) by applying external perturbations, *i.e.*, strain and electric field, results in multi-functional devices that may be utilized as transistors and optoelectronic devices. When contemplating the numerous conceivable configurations of 2D and TMDCs materials, there are very few experimental studies of applying external perturbations to integrating 2D TMDCs with other 2D materials. More study is needed to conceptualize and build unique heterostructures made up of various 2D and TMDCs materials, which might lead to some unexpected properties and applications.

(ii) Most TMDC-based heterostructures have type II band alignment, which has been reported theoretically or experimentally, and just a handful have type III band alignment. In this work, we propose a simple yet effective technique for converting type II band alignment heterostructures to type III or *vice versa* by using biaxial strain and an electric field. Type II heterostructures, for example, can be used in optoelectronics, whereas type III heterostructures can be used as FETs. When external strain or an electric field is applied to 2D(TMDCs)/TMDCs(2D) heterostructures, both type II and type III band alignment is achieved, resulting in multi-functional characteristics.

Reviewing recent advances in TMDC-based heterostructures, we demonstrated a plethora of alternative possibilities for innovative multi-functional devices, enabled by the capacity to modify the band structure in totally new ways compared to traditional 3D semiconductors.

## Author contributions

All authors contributed to writing the manuscript, overall draft preparation, review, and editing. All authors have read and agreed to the published version of the manuscript.

## Conflicts of interest

There are no conflicts to declare.

## Supplementary Material
